# Quantitative trait loci (QTL) analysis of leaf related traits in spinach (*Spinacia oleracea* L.)

**DOI:** 10.1186/s12870-021-03092-5

**Published:** 2021-06-24

**Authors:** Zhiyuan Liu, Hongbing She, Zhaosheng Xu, Helong Zhang, Guoliang Li, Shifan Zhang, Wei Qian

**Affiliations:** Institute of Vegetables and Flowers, Chinese Academy of Agricultural Sciences, Beijing, China

**Keywords:** Spinach, KASP, Leaf-related traits, Genetic linkage map

## Abstract

**Background:**

Spinach (*Spinacia oleracea* L.) is an important leafy vegetable crop, and leaf-related traits including leaf length, leaf width, and petiole length, are important commercial traits. However, the underlying genes remain unclear. The objective of the study was to conduct QTL mapping of leaf-related traits in spinach.

**Results:**

A BC_1_ population was used to construct the linkage map and for QTL mapping of leaf length, leaf width, petiole length, and the ratio of leaf length to width in 2015 and 2019. Two genetic linkage maps were constructed by specific locus amplified fragment sequencing (SLAF-seq), and kompetitive allele specific PCR (KASP) technology, respectively using BC_1_ population in 2015. Based on the results of 2015, the specific linkage groups (LG) detected QTLs were generated using BC_1_ population in 2019. A total of 13 QTLs were detected for leaf-related traits, only five QTLs being repeatedly detected in multiple years or linkage maps. Interestingly, the major QTLs of leaf length, petiole length, and the ratio of leaf length to width were highly associated with the same SNP markers (KM3102838, KM1360385 and KM2191098). A major QTL of leaf width was mapped on chromosome 1 from 41.470−42.045 Mb. And 44 genes were identified within the region. Based on the GO analysis, these genes were significantly enriched on ribonuclease, lyase activity, phosphodiester bond hydrolysis process, and cell wall component, thus it might change cell size to determine leaves shape.

**Conclusions:**

Five QTLs for leaf-related traits were repeatedly detected at least two years or linkage maps. The major QTLs of leaf length, petiole length, and the ratio of leaf length to width were mapped on the same loci. And three genes (*Spo10792, Spo21018*, and *Spo21019*) were identified as important candidate genes for leaf width.

**Supplementary Information:**

The online version contains supplementary material available at 10.1186/s12870-021-03092-5.

## Background

Spinach (*Spinacia oleracea* L.) is an economically significant leafy vegetable that is grown worldwide. It is also regarded as one of the most nutritious vegetables due to its high quantity of substances that are beneficial to human health, including vitamins A, E, C, K, lutein, folic acid, oxalic acid, calcium, iron phosphorous and potassium [1–3]. Spinach originated from middle-east (possibly Iran) and has been cultivated for over two thousand years. China began cultivating spinach during the seventh century, which is earlier than Europe and United States [4]. China is the largest producer, followed by USA and Japan [2]. In 2014, the global production of spinach is estimated to be approximately 24.3 million tones, 91 % of which is produced in China [5].

Spinach is a diploid species which belongs to the Amaranthaceae family and commonly defined as dioecious, but there are occasionally monoecious species [6, 7]. Leaf traits such as leaf type (savoy, semi-savoy, and smooth type), leaf length and width, leaf erectness, petiole length and color, and edge shape, are regarded as important agronomic characteristics making a significant impact on spinach breeding. Erect leaves, for example, are more likely to accommodate high-density spinach production and mechanical harvesting. Additionally, savoy and semi-savoy leaf types of spinach are generally suitable for fresh-market consumption while smooth types are prevalent for freeze products and salad [3, 8]. Such traits are typical quantitative traits controlled by multiple genes and can also be significantly influenced by environmental conditions [9]. Until now, only a few reports have been published associating leaf-related traits with molecular markers in spinach. Ma et al. (2016) [3] performed association mapping of surface texture, edge shape, and petiole color of spinach using genotyping-by-sequencing (GBS) technology and found 5, 7, and 14 single nucleotide polymorphism (SNP) markers were associated with surface texture, edge shape, and petiole color, respectively. More recently, Cai et al. (2018) [5] used GBS technology and QTL mapping on leaf color (red/green). The authors found that one major QTL accounted for 69.3 % of phenotypic variation. However, the QTLs of leaf type (length and width) and petiole length are still unclear. Thus, mapping of the QTLs could have a significant influence on spinach improvement.

Genetic linkage maps are a powerful genomic tool, which have been widely used for QTL mapping, gene mapping, whole-genome assembly, and marker-assisted selection (MAS) [10]. To date, five genetic linkage maps of spinach have been reported. The first genetic map contained 101 amplified fragment length polymorphism (AFLP) and 9 simple sequence repeats (SSR) that were generated using PCR-based markers [11]. This map was divided into seven linkage groups, spanning 585 cM, with an average of 5.18 cM between adjacent markers, and it was used for analyzing the location of a sex-determining locus [11]. The second genetic map was 433.6 cM and was generated with 283 SNP markers using RNA sequencing [12]. The map was divided into six linkage groups, consistent with the number of spinach chromosomes, and 39 QTLs associated with nitrogen use efficiency in spinach were identified using the linkage map [12]. The third genetic map was constructed using specific locus amplified fragment sequencing (SLAF-seq) technology and 4,080 SNP markers and spanning 11,125.97 cM, with an average distance of 0.31 cM between the adjacent markers [13]. The fourth genetic map was constructed by Xu et al. (2017) [14], and it contained six linkage groups, and 463 Mb spinach genome scaffolds were found to be anchored to the genetic map with 870 SNP markers. Recently, Cai et al. (2018) [5] constructed an updated high-density genetic map to map leaf color (ref/green). The map contained six linkage groups spanning 1539.96 cM, and average marker interval was 0.4 cM, as measured using GBS technology.

The type and number of polymorphic DNA markers play an important role in constructing a high- density genetic linkage map. Until now, various types of DNA markers have been discovered, including restriction fragment length polymorphism (RFLP), randomly amplified polymorphic DNA (RAPD), AFLP, SSR, and SNP [15, 16]. SNP are the most common type of DNA markers, which have a high abundance and can support cost-effectiveness and rapid screening processing [17]. KASP is a PCR-based homogeneous fluorescent SNP genotyping technology that can discriminate allele gene hinge on different fluorescent (e.g., FAM and HEX fluorescent) [18]. With the advantages of cost-effectiveness, adaptability, and efficiency, this technology is widely used on many species, including wheat [19], tomato [20], and maize [21].

In the study, high-density spinach genetic map [13] was used to map the QTLs of leaf length (LL), leaf width (LW), petiole length (PL), and the ratio of leaf length to width (LR). Additionally, in order to verify the authenticity of SLAF markers, we constructed a genetic linkage map using SLAF markers that was based on KASP technology and attempted to use the linkage map to map the four traits. Our objective was to develop DNA molecular markers for MAS in spinach that could be used for the improvement of the crop and future research on spinach.

## Results

### Identification of SNP markers and genotyping

**Fig. 1:**
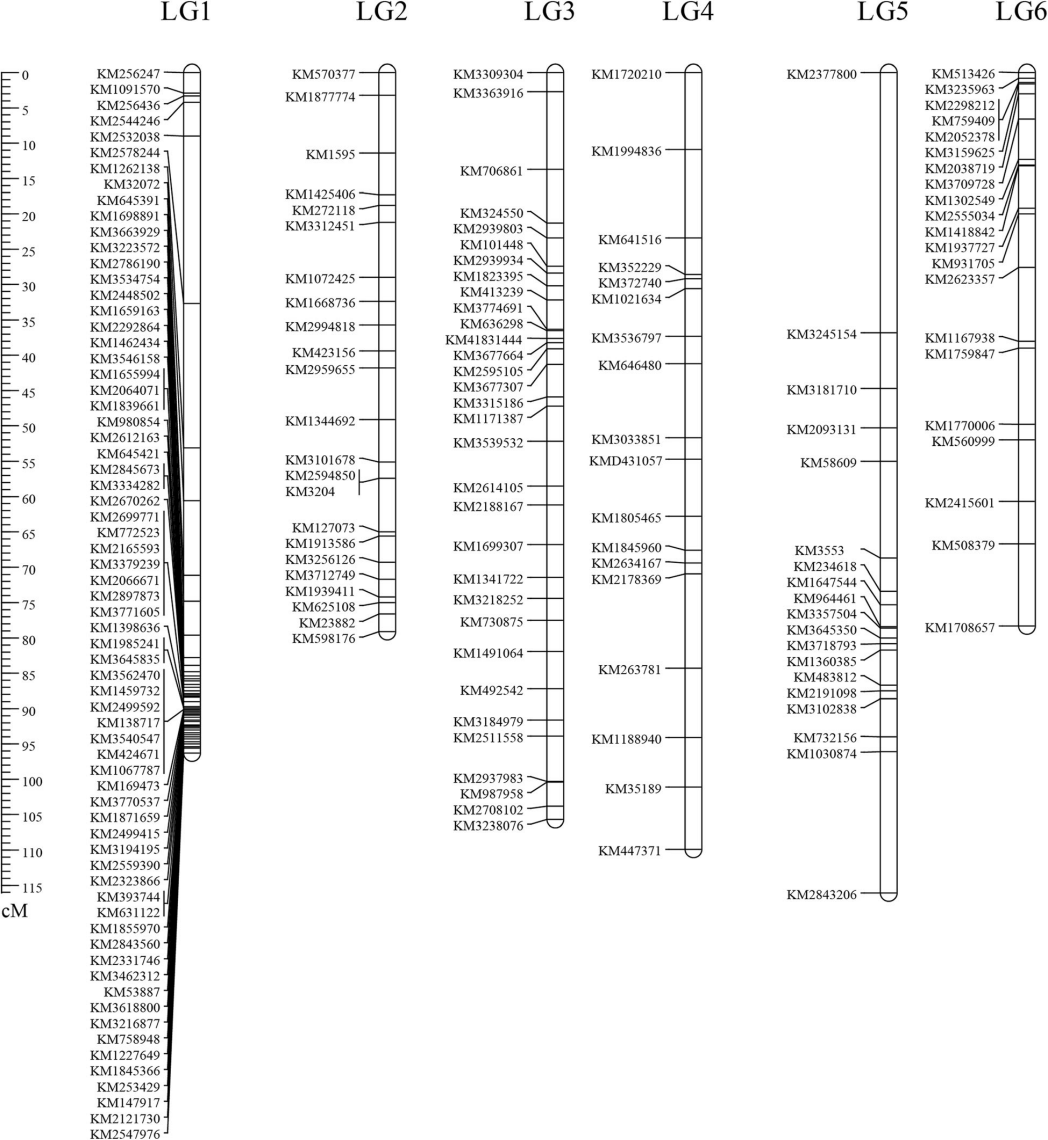
Distribution of markers in six linkage groups in the BC_1_ population from 2015. LG1-LG6 represent six putative linage groups (LG). Markers names are shown left in each LG and the ruler indicates map coordinates (cM)

In order to validate the accuracy of 4,080 SLAF markers from SLAF-seq technology (Table S1) [13], we evenly chosen 300 SLAF markers from the six high-density linkage groups to construct a linkage map with evenly-distributed markers that were based on KASP technology. The markers were used to genotype the BC_1_ individuals. Of these 300 markers, 119 markers were not available due to a lack of segregation, distorted segregation, or signal. Therefore, the remaining 181 available markers were used to generate linkage map (Fig. 1).

### Construction of linkage map

**Fig. 2:**
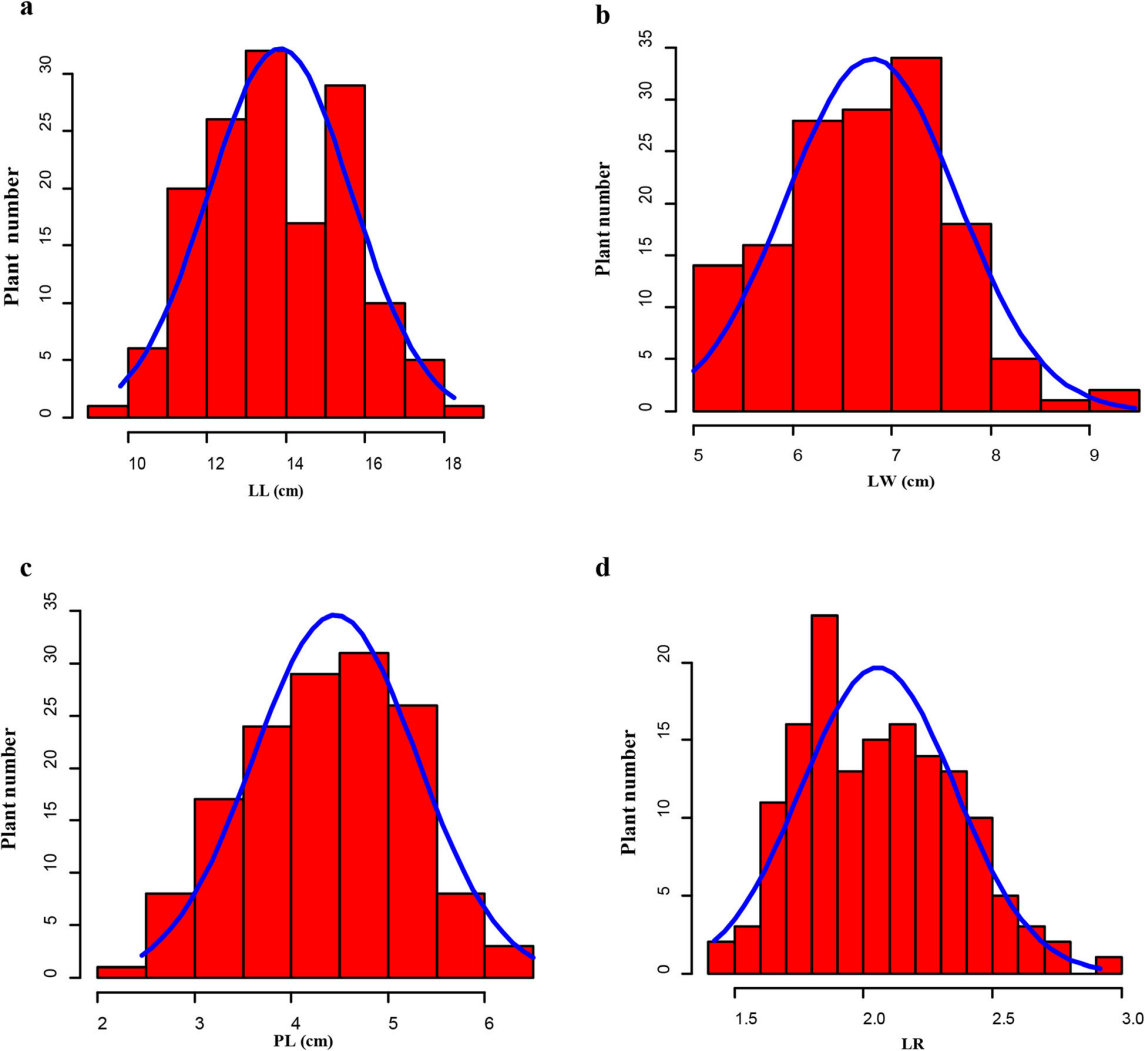
QTLs results of LL, LW, PL, and LR using high-density genetic linkage map. Red lines indicate the threshold value; red arrows represent potential QTLs

**Fig. 3:**
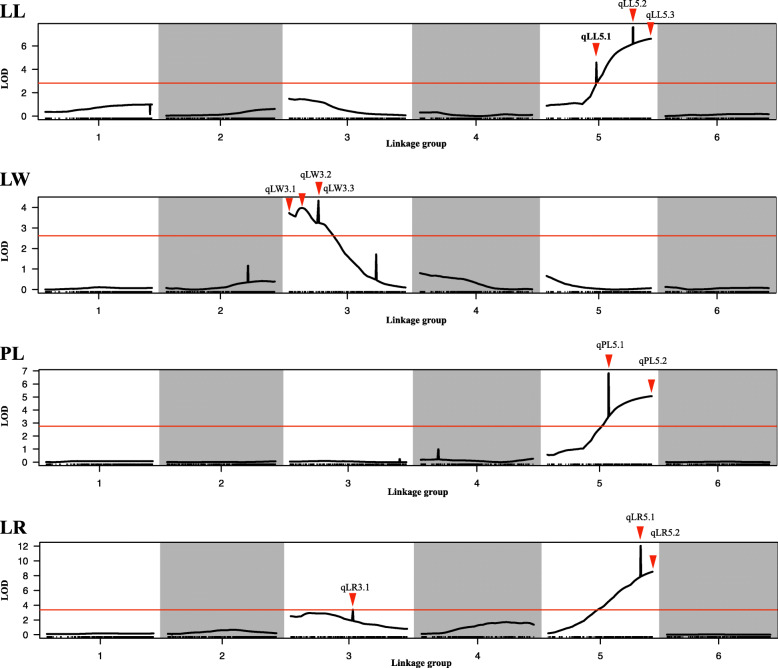
QTLs results of LL, LW, PL, and LR using BC_1_ population from 2015 and 2019, respectively. The red indicates the results from 2015 while blue from 2019

To develop an SNP-based genetic linkage map, the 181 informative SNP markers and the 147 BC_1_ individuals from 2015 were used for linkage analysis using JoinMap4.0 software. All of the markers were mapped onto the genetic map that was divided into six linkage groups (LG1 to LG6), which is consistent with the number of spinach chromosomes (Fig. 1 and Table S2). The total size of the linkage map was 585.45 cM with an average interval of 3.23 cM. The linkage group containing the maximum numbers of markers was LG1, where there were 68 SNP markers, and it spanned 96.29 cM. The LG4 had a length of 109.911 cM and included 18 markers, the minimum number of markers among the six linkage groups. Some gaps existed in LG1, LG4 and LG5 due to a lack of enough evenly-distributed markers, but the linkage groups with evenly-distributed markers also observed like LG2 and LG3. Based on the results of QTLs using BC_1_ population from 2015 (Figs. 2 and 3), the markers located on the LG3 and LG5 were used to generate genetic map and attempt to map the four traits using BC_1_ population from 2019 (Table S3).

### Analysis of phenotypic data of BC_1_ population

**Fig. 4:**
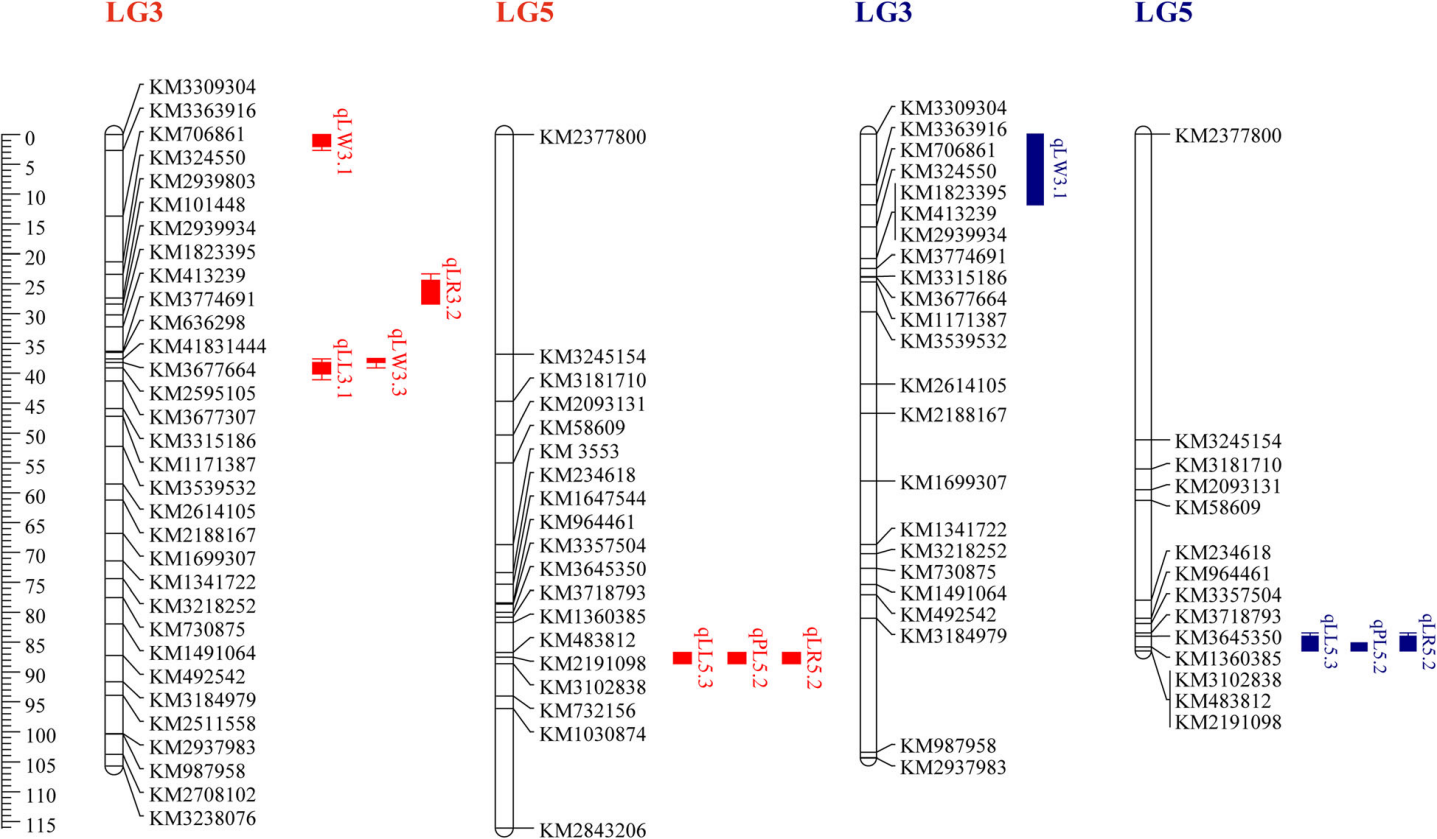
Frequency distributions of spinach leaf-related traits in BC_1_ population from 2015

Leaf-related traits of BC_1_ lines are summarized in Table 1. As for the individuals in 2015, the LL varied widely, ranging from 9.80 to 18.25 cm, with an average of 13.85 cm. LW ranged from 5.00 to 9.45 cm, with a mean of 6.80 cm. PL ranged from 2.45 to 6.50 cm, with an average of 4.45 cm. The BC_1_ lines in 2019 shared higher value of LL, LW, and PL relative to the individuals in 2015 (Table S4). Frequency distributions of LL, LW, PL, and LR in the BC_1_ population from 2015 to 2019 had a continuous distribution (Fig. 4a, b, c, d; Fig. S1a, b, c, d), representing four traits controlled by multiple genes. In addition, LL was significantly and positively correlated to PL (*r* = 0.82) among the BC_1_ population in 2015 but correlated with LW (r = 0.73) in 2019 (Table 2), which may be affected by the environment.

**Table 1:** Phenotypic variation of leaf-related traits for two parents and BC_1_ lines

Traits		Parents		BC_1_ lines					
(cm)	Years	12S3	12S4	Min	Max	Mean±SE	Skewness	Kurtosis	CV%
LL	2015	11.50	17.00	9.80	18.25	13.85±1.82	0.12	-0.68	13.14 %
	2019	13.0	15.2	10.45	22.9	15.44±0.17	0.49	0.20	14.81 %
LW	2015	7.25	6.00	5.00	9.45	6.80±0.86	0.10	0.07	12.655
	2019	11.33	9.80	8.25	17.75	11.45±0.12	0.79	1.23	13.60 %
PL	2015	3.50	6.50	2.45	6.50	4.45±0.85	-0.02	-0.65	19.09 %
	2019	5.28	9.81	3.25	12.9	8.46±0.14	0.15	-0.37	22.61 %
LR	2015	1.92	2.34	1.42	2.92	2.06±0.30	0.35	-0.41	14.49 %
	2019	1.26	1.47	1.06	1.98	1.34±0.01	0.78	1.63	10.25 %

*SE* standard error; *CV* coefficient of variation

**Table 2:** Correlation coefficients of four leaf-related traits in 2015 and 2019

Traits in 2015	LL	PL	LW
PL	0.82		
LW	0.38	0.18	
LR	0.57	0.59	-0.52
Traits in 2019	LL	PL	LW
PL	0.42		
LW	0.73	0.17	
LW	0.46	0.38	-0.26

### QTL mapping for leaf-related traits

Based on LOD threshold for the LL (2.8), LW (2.6), PL (2.8), and LR (3.4), 13 QTLs for the four traits were detected and were found to be distributed on LG3 and LG5 using three linkage maps: high-density genetic map generated by SLAF-seq (Qian et al. 2017), genetic map constructed by BC_1_ population from 2015 to 2019 using KASP technology, respectively. (Table 3) (Figs. 2 and 3).

**Table 3:** QTL analysis of spinach leaf-associated traits

Traits	QTL	Linkage maps		Closed Marker	Position (cM)	Marker interval	LOD	% Expl.	Add.
LL	qLL5.1	SLAF-seq		Marker3744695	86.036	Marker236827- Marker1233484	4.58	6.1	1.69
	qLL5.2		SLAF-seq	Marker3268124	149.824	Marker1984033- Marker628287	7.60	20.1	2.02
	qLL5.3		SLAF-seq	Marker3102838	180.406	Marker600011- Marker3102838	6.61	14.2	1.93
	qLL5.3		2015	KM2191098	87.503	KM1360385- KM3102838	24.58	50.7	2.58
	qLL3.1		2015	KM2595105	39.139	KMC1_4183144- KM3677307	3.7	5.4	0.84
	qLL5.3		2019	KM1360385	76.692	KM3718793- KM3102838	8.39	22.2	2.19
LW	qLW.3.1		SLAF-seq	Marker3309304	0	Marker3309304- Marker2310789	3.72	6.8	0.71
	qLW.3.2		SLAF-seq	Marker3098699	22.339	Marker3109716- Marker40134	3.97	7.2	0.78
	qLW.3.3		SLAF-seq	Marker3677664	50.675	Marker351364- Marker643757	4.434	9.3	0.76
	qLW.3.1		2015	KM3309304	0	KM3309304- KM3363916	3.01	6.9	0.51
	qLW.3.3		2015	KM41831444	37.623	KM636298- KM2595105	3.9	9	0.57
	qLW.3.1		2019	KM3309304	4	KM3309304- KM706861	2.02	5.7	0.80
PL	qPL5.1		SLAF-seq	Marker3412457	105.929	Marker1572269- Marker3601009	6.82	19.5	0.77
	qPL5.2		SLAF-seq	Marker3102838	180.406	Marker3601009- Marker3102838	5.06	16.5	0.67
	qPL5.2		2015	KM2191098	87.503	KM483812- KM3102838	12.14	31.6	0.94
	qPL5.2		2019	KM3102838	77.315	KM3645350- KM2191098	9.28	24.2	1.84
LR	qLR3.1		SLAF-seq	Marker3339555	107.483	Marker304732- Marker3743797	3.39	6.9	-0.11
	qLR5.1		SLAF-seq	Marker1997127	159.994	Marker376664- Marker870652	12.03	25.1	0.31
	qLR5.2		SLAF-seq	Marker3102838	180.406	Marker870652- Marker3102838	8.53	20.3	0.31
	qLR3.2		2015	KM101448	27.414	KM2939803- KM2939934	4.36	6.4	-0.1
	qLR5.2		2015	KM2191098	87.503	KM1360385- KM3102838	24.76	51.4	0.42
	qLR5.2		2019	KM1360385	76.692	KM3718793- KM3102838	14.43	32.5	0.17

*% Expl*, Percentage of phenotype variation explained; *Add.* additive effect

For the LL, four QTLs (qLL5.1, qLL5.2, 1LL5.3, and qLL3.1) were identified and were distributed on LG5 and LG3. Only qLL5.3 was repeatedly detected on the three linkage maps and had the LOD score ranging from 6.61 to 24.58 and could explain 14.2−50.7 % of the phenotypic variation. Based on the results from the three linkage maps, three closest markers, Marker3102838, KM2191098, and KM1360385, were located on 76.692−77.340 cM region significant associated with qLL5.3 (Fig. 3).

Three QTLs for LW were found: qLW3.1, qLW3.2, and qLW3.3. qLW3.3, a major QTLs, was repeatedly detected on two linkage maps in 2015 while a minor QTL qLW3.1 was repeatedly found on the three linkage maps. The qLW3.3, close to two adjacent markers Marker3677664 and KM41831444, had the LOD score of 3.9−4.434 and explained 9−9.3 % of the phenotypic variation. The minor LW QTLs, close to marker KM3309304 (Marker3309304), had an LOD score of 2.02−3.72 and explained 5.7−6.9 % of the phenotypic variation.

Only two QTLs (qPL5.1, and qPL5.2) were found for PL, only qPL5.2 was repeatedly identified on the three linkage maps and it contained an LOD score of 5.06−12.14. This QTL (qPL5.2) was detected on LG5 and had a peak at two adjacent markers KM2191098 and KM3102838 (Marker3102838), and explained 16.5−31.6 % of the phenotypic variation and 0.67−1.84 additive effect.

For the LR, four QTLs (qLR5.1, qLR5.2, qLR3.1, and qLR3.2) were found. qLR5.2 was found to be a major QTL with an LOD score of 8.53−14.76 and repeatedly detected on the three linkage maps. Interestingly, qLL5.3, qPL5.2, and qLR5.2 were detected on three linkage maps and mapped on the same locus of LG5 close to three adjacent markers KM1360385, KM2191098, and KM3102838.

**Fig. 5:**
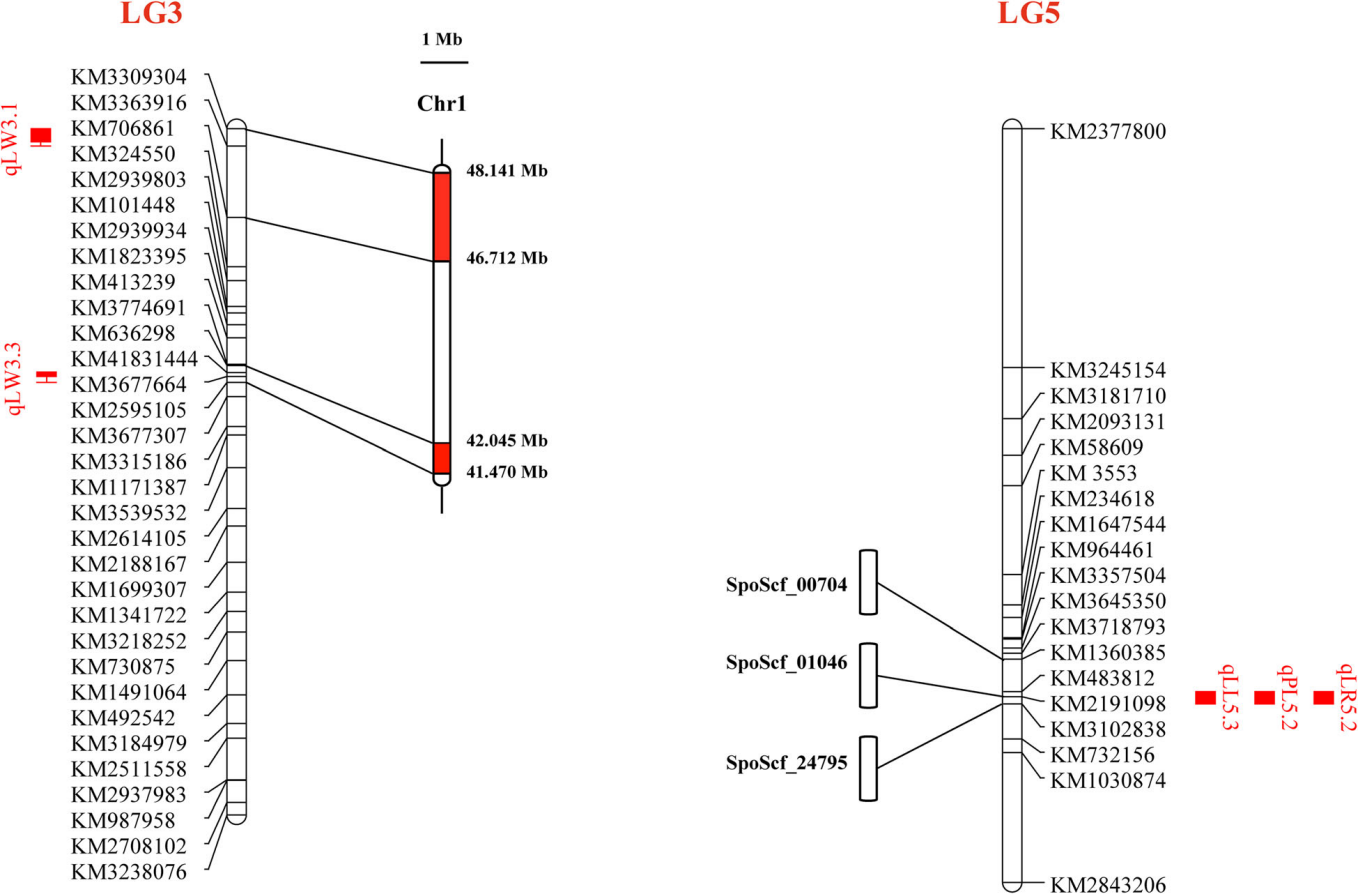
The physical position of the QTLs that were detected at least on two linkage maps

The three major QTLs (qLL5.3, qPL5.2, and qLR5.2) were highly associated with KM1360385, KM2191098, and KM3102838, which were located on three scaffolds, SpoScf_00704 (321,888 bp), SpoScf_01046 (226,571 bp), and SpoScf_24795 (1428 bp), respectively. The major QTL qLW3.3 was mapped on 41.470−42.045 Mb and the minor QTL qLW3.1 was mapped on 46.712−48.141 Mb (Fig. 5). The above spinach reference database was from SpinachBase (http://www.spinachbase.org).

### Prediction and analysis of candidate genes

**Fig. 6:**
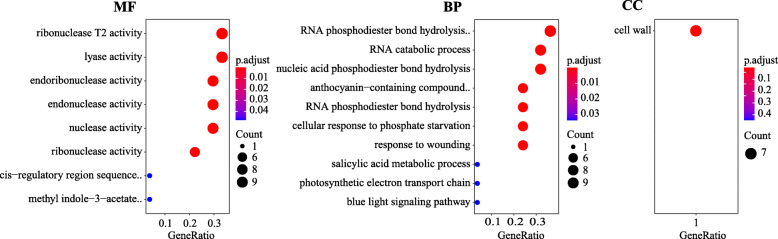
The annotation of the 44 genes in the 41.470-42.045 Mb on chromosome 1 through GO analysis. MF: molecular function, BP: biological process, CC: cellular component (*p* value < 0.05)

Based on the genome annotation information, the genes within the QTL confidence intervals could be identified. For the LL, LR and PL locus, two genes and nine genes were identified in scaffold SpoScf_01046 and SpoScf_00704, respectively, while no genes were found in SpoScf_24795 (Table S5). All of the genes encoded BNR/Asp-box repeat family protein, Leucine-rich repeat, protein kinase, etc. (Table S5). As for the LW, 44 genes were found of the major QTL qLW3.3 locus that was located on chromosome 1 from 41.470 to 42.045 Mb (Table S6). In GO analysis, 27 out of the 44 genes were enriched in the molecular function (MF) category, 25 genes were enriched in the biological process (BP) category, and only seven gene were found in cellular component (CC) category (*p* value < 0.05). In the MF category, most genes were related to ribonuclease T2 activity, lyase activity, and endonuclease activity etc. (Fig. 6). In the BP category, most genes were involved in RNA phosphodiester bond hydrolysis, endonucleolytic, RNA catabolic process, and nucleic acid phosphodiester bond hydrolysis. In the CC category, the seven genes were related to cell wall (Fig. 6). Based on the annotation information of spinach reference, three genes (*Spo10792, Spo21018*, and *Spo21019*) were identified because they are involved in cell expansion (Table 4).

**Table 4:** Important genes associated with leaf width within 41.470–42.045 Mb on chromosome 1

Genes	Physical position (Mb)	Annotation
*Spo10792*	41.75	Causes loosening and extension of plant cell walls
*Spo21018*	42.02	NAC domain protein, multidimensional cell growth
*Spo21019*	42.03	NAC domain protein, multidimensional cell growth

### Development of marker linked to the leaf-related traits

**Fig. 7:**
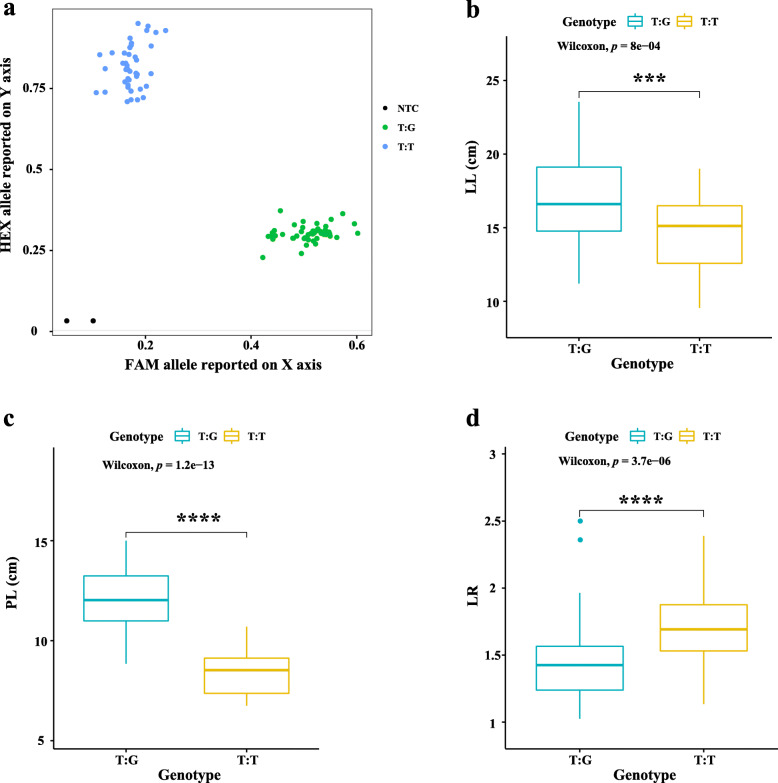
Ninety BC_1_ individuals in 2020 were used for validation the marker KM2191098 closely linked to LL, PL, and LR. (**a**) Genotype plot for 90 individuals using the KASP marker KM2191098; Wileoxon test was conducted between genotype T:G and T:T in LL (**b**), PL (**c**), and LR (**d**)

In the present study, the SNPs tightly linked to the four major QTLs, qLL5.3, qPL5.2, qLR5.2 and qLW3.3, were able to develop KASP markers for MAS. For example, 90 BC_1_ individuals planted in 2020 were used to examine the accuracy of marker KM2191098, which is associated with the LL, PL, and LR. As shown in Fig. 7a, KASP genotype assay analysis (see “Materials and methods”) showed that the 90 lines could be obviously separated two groups: T:G and T:T genotype, indicating that KM2191098 is an available marker. And the detailed information of genotype and phenotype were summarized in Table S7. The primer sequence of marker KM2191098 was summarized in Table S8. Otherwise, comparison of the phenotype between T:G and T:G in LL, PL, and LR, the two genotypes shared significant difference in the three traits (Fig. 7b, c, d). The individuals with T:G genotype, for instance, shared longer leaf length in comparison to the T:T. Therefore, these results demonstrated that the marker KM2191098 linked to the LL, PL, and LR could be used for MAS.

## Discussion

Leaf traits are very important because they affect how leaves capture light for photosynthesis [22]. Until now, there have not been many studies related to leaf or petiole traits in spinach [3, 9, 23]. However, QTLs associated with LL, LW, and PL in spinach remain unreported.

In the study, a high-density genetic map constructed by Qian et al. 2017 and two genetic maps were generated using KASP genotyping assay were used to map QTLs for LL, LW, PL, and LR. We identified four QTLs associated with LL (qLL5.1, qLL5.2, 1LL5.3, and qLL3.1), three QTLs associated with LW (qLW3.1, qLW3.2, and qLW3.3), two QTLs related to PL (qPL5.1, and qPL5.2), and four QTLs for LR (qLR5.1, qLR5.2, qLR3.1, and qLR3.2). Interestingly, the qLL5.3, was located in the same region as the major QTLs qPL5.2 and qLR5.2, suggesting the LL is correlated with PL or LR, which is consistent with the correlation in Table 2. Among them, only five QTLs (qLL5.3, qPL5.2, qLR5.2, qLW3.3, and qLW3.1) were repeatedly detected at least on two linkage maps (Figs. 2 and 3). Two reasons were considered to account for the fact that only minority QTLs were able to detect at different linkage maps. On the one hand, the differences in QTL numbers, position might be arisen from environments effects. On the other hand, different population, linkage maps, and even the number of markers could also affect QTLs [24, 25]. For example, the QTL qLR5.1 was only detected on the high-density linkage map while qLR5.2, close to qLR5.1, was able to be repeatedly detected on the three different linkage maps (Figs. 2 and 3), thus we speculated that the two QTLs might be the same one. Therefore, these QTLs were not able to be repeatedly detected in multiple years or linkage maps.

Until now, many genes involved in determining leaf shape and size have been reported. For example, in *Arabidopsis thaliana*, brassinosteroid-responsive RING-H2 (*BRH1*) gene, contains highly conserved RING finger domain and was able to change the leaves shape [26]. Big brother (*BB*) was able to greatly reduce leaf size [27]. *WOX5*, one of the WUSCHEL-related homeobox family, could acts redundantly with *WOX1* and *WOX3* to control leaf shape [28]. Many investigations on leaf shape and size have also been conducted in rice. NARROW LEAF 7 (*NAL7*) encodes a flavin-containing mono-oxygenase and results in a reduced leaf width mediated by auxin [29]. Narrow and Rolled Leaf 1 (*NRL1*) encodes the cellulose synthase-like protein D4, and Hu et al. (2010) identified three recessive mutants (*nrl1-1, nrl1-2, and nrl1-3*) with the narrow-rolled leaf phenotype [30]. Qi et al. (2008) characterized a mutant named narrow leaf1 (*nal1*), which reduced leaf width by affecting vein patterning and polar auxin transport [31]. Narrow-curly leaf genes *NAL2* and *NAL3* encoded a WUSCHEL-related homeobox transcriptional activator and controlled leaf width in rice [9].

Taken together, the genes involved in organ growth, development, or hormone transport are likely to change the leaves shape. In the present study, the major QTLs of LL, PL, and LR were mapped on three scaffolds (SpoScf_00704, SpoScf_24795 and SpoScf_01046) as the incompleteness of the spinach genome assembly. Eleven genes were identified from the three scaffolds and detailed information were list in Table S5. Moreover, the major QTL qLW3.3 was located at 41.470−42.045 Mb on chromosome 1, and 44 genes were identified within the region (Table S6). According to the GO analysis, the 44 genes were mainly enriched on ribonuclease, lyase activity, phosphodiester bond hydrolysis, and cell wall (Fig. 6), thus we hypothesized that the leaf width might be changed due to cell expansion as previously described that cell expansion can determine leaf final size and shape after their basic shape is established [32]. Furthermore, based on the annotation of the spinach reference genome (http://www.spinachbase.org), three genes (*Spo10792*, *Spo21018*, and *Spo21019*), involved in cell expansion, were selected as potential candidate genes (Table 4). Further work is required to verify this result.

In recent years, molecular markers were prevalent for many crop breeding programs due to it could improve breeding efficiency and accelerate breeding progress. Therefore, MAS is an efficient and effective approach for breeders [33]. In the present study, based on the four major QTLs, qLL5.3, qPL5.2, qLR5.2 and qLW3.3, we could develop effective KASP markers for MAS. For example, the KASP markers KM2191098, which is associated with the LL, PL, and LR (Fig. 7). Therefore, SNP tightly linked to the four traits would be valuable for screening interest leaf shape and size in spinach.

## Conclusions

Thirteen QTLs for spinach leaf-related traits were identified using SLAF-seq and KASP technology. And only five QTLs were repeatedly detected in multiple years or linkage groups. The major QTL qLW3.3 was mapped on chromosome 1 from 41.470 to 42.045 Mb. Forty-four genes were identified within the candidate region and three genes (*Spo10792, Spo21018*, and *Spo21019*) were considered to be potential candidate genes, which were involved in growth regulation or cell expansion. Interestingly, three major QTLs qLL5.3, qPL5.2, and qLR5.2 were highly associated with KM1360385, KM2191098, and KM3102838. Based on the results, one KASP marker was developed, which was significantly related to LL, PL, and LR. The findings in this study would be valuable for leaf-related traits breeding program and further fine mapping the traits in spinach.

## Methods

### Plant materials and DNA extraction

**Fig. 8:**
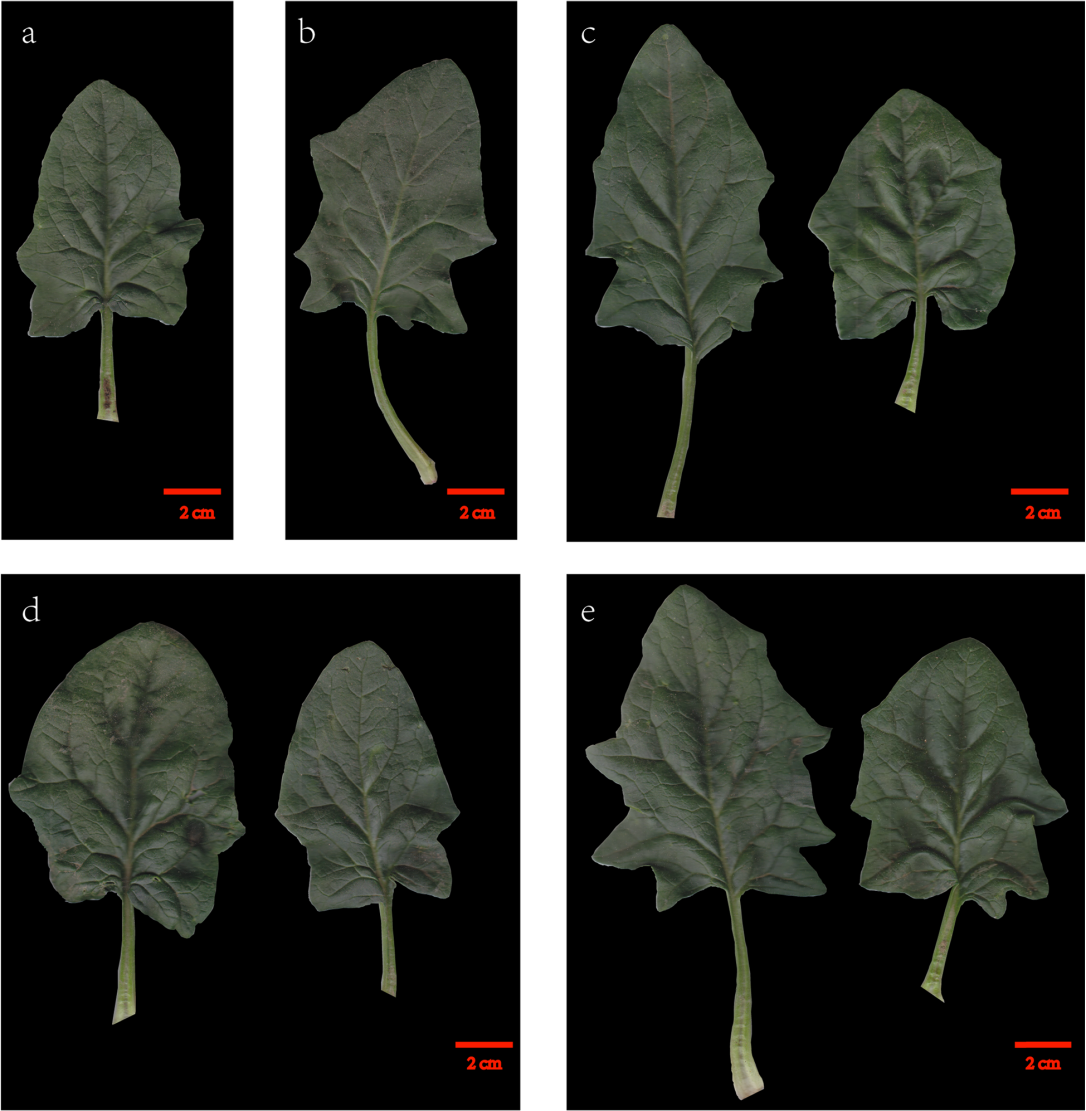
Plant materials used in the study: (**a**) female parent; (**b**) male parent; and (**c**), (**d**), and (**e**) contrasting leaf length, leaf width, and petiole length in BC_1_ population from 2015

A 147 BC_1_ mapping population was derived from across between the inbred line 12S3, a female and recurrent parent, and a male parent (inbred line 12S4). All the plant materials were generated by the spinach research group of the Institute of Vegetables and Flowers (IVF), Chinese Academy of Agriculture of Sciences (CAAS). Compared with the inbred line 12S3 (Fig. 8a), 12S4 contained a larger leaf and petiole length but smaller leaf width (Fig. 8b). The entire BC_1_ population (Fig. 8c−e) and parents were cultivated in a natural environment in the experimental field of the IVF, CAAS in the spring of 2015 and 2019, respectively. Additionally, 90 BC_1_ individuals used for validation the marker closely linked to leaf related traits were also planted in the experimental field of the IVF in the spring of 2020. All above individual plants were spaced 0.3 m apart and with row spacing of 0.5 m in a plot. Otherwise, the plant materials used and collected in the study comply with China’s guidelines and legislation.

The fresh leaves of BC_1_ individuals and two parents (until 6 true leaves emerged) were collected and then frozen in liquid nitrogen until DNA extraction. Total genomic DNA was extracted using the cetyltrimethyl ammonium bromide (CTAB) method as described by Murray and Thompson (1980) [34]. The DNA quality and concentration were assessed using electrophoresis in 1.0 % agarose gels and ND-2000 spectrophotometer (Thermo Fisher Scientific, Wilmington, DE, USA).

### Discovery of SNP molecular markers and genotyping

A total of 4,080 polymorphic SLAF markers were identified through the SLAF-seq for the two parents, using methods as previously described by Qian et al. (2017). To verify the accuracy of SLAF markers using KASP technology, 300 SLAF markers, renamed “KM”, followed by the SLAF markers number, were selected to design the KASP assay primer, and primers were designed by the LGC company (Shanghai, China). KASP was performed in a volume 5 µL, including 2.5 µL 2 × KASP Master mix, 0.07 µL KASP Assay mix, and 2.5 µL DNA that was diluted to 20−30 ng/µL. The KASP genotyping mixes were arrayed into a 384-well PCR plate. No-template controls (NTCs) were included on each plate.

All amplifications were performed on a Veriti 384 Well Thermal Cycler (Applied Biosystems, Foster City, CA, US) using the following thermal cycling conditions: 15 min at 94 ° C followed by thermal cycles of 20 s at 94 ° C and 60 s at 61 ° C (drop 0.6 ° C per cycle), 26 cycles of 20 s at 94 ° C and 60 s at 55 ° C. Next, the plate was read below 40 ° C in QuantStudio 12 K Flex Real-Time PCR System (Applied Biosystems, Foster City, CA, US). If sufficiently defined genotype clusters are not obtained after the initial KASP thermal cycles, an additional thermal cycle is required (three cycles of 20 s at 94 °C and 60 s at 57 °C). The additional thermal cycling and plate reading was performed until defined genotype clusters were attained.

### Construction of linkage map

Markers were filtered out at segregation distortion, and then the available SNP markers were subjected to linkage analysis and map construction using BC_1_ population from 2015 by JoinMap 4.0 software [35]. The regression mapping method was used to calculate each linkage group to order the markers. The start value of the independence LOD was changed to 0.5, and all other settings were set to the default values. As for the BC_1_ population from 2019, the SNP markers located on linkage group with QTLs identified using individuals from 2015 and high-density genetic map were selected to construct genetic map to further map the four traits.

### Phenotypic evaluation

Phenotypic data were collected from BC_1_ individuals in 2015, 2019, and 2020. The LL, LW, and PL of outer two leaves were measured using a ruler once the individuals contained six true leaves. Frequency distribution and correlation of the LL, LW, PL, and LR were plotted using R software (version 3.5.1).

### QTL analysis and candidate genes identification

QTLs for the four leaf related traits were detected via three different linkage maps: high-density genetic map generated by SLAF-seq [13], genetic map constructed by BC_1_ lines from 2015 to 2019, respectively. QTL analysis was performed with MapQTL 6.0 [36] using interval mapping (IM) and the multiple QTL model (MQM) methods. The general process was (1) the LOD threshold, essential parameter for QTL, was calculated using the permutation test at the genome-wide significance level of P < 0.05, n = 1000; (2) QTL mapping was conducted using the IM method with the set step size as 1; (3) according to the result of IM mapping, the markers closest to the LOD peak that were higher than the threshold were obtained as cofactors, and MQM mapping analysis was conducted; (4) the markers nearest to the LOD peak were examined, and the set of cofactors were adjusted correspondingly. After the fourth step, we repeated the MOM mapping. (5) The markers closest to the highest LOD score were considered the QTL, and a 1.5-LOD support interval was constructed as confidence intervals by selecting the region that the LOD score on either side of the peak decreases by 1.5 LOD. We used standard QTL nomenclature: the first letter of the ‘q’ and trait, followed by the name of linkage group, followed by the number of QTL on the same linkage group (e.g., qLW3.2 for the second QTL of leaf width on the linkage group 3).

The QTLs were repeatedly detected at least two linkage maps were defined as the valuable QTLs. The confidence intervals from three linkage maps were merged and then considered as the finally confidence intervals. The candidate genes were categorized through gene ontology (GO) analysis using clusterProfiler [37]. The boxplots were performed using BMK Cloud platform (http://www.biocloud.net/).

## Supplementary Information

**Additional file 1:Table S1.** The information of 4080 SLAF markers in 147 individuals of BC_1_ population from 2015 (XLSX).

**Additional file 2:Table S2.** The information of 181 KASP markers in 147 individuals of BC_1_ population from 2015 (XLSX).

**Additional file 3:** **Table S3.** The information of 37 KASP markers in 147 individuals of BC_1_ population from 2019 (XLSX).

**Additional file 4:** **Table S4.** Phenotype of the four leaf-related traits of BC_1_ population from 2015 to 2019 (XLSX).

**Additional file 5:** **Table S5.** The number of genes on the three scaffolds related to LL, PL and LR (XLSX)

**Additional file 6:Table S6.** The number of genes within the region on chromosome 1 from 41.470 to 42.045 Mb (XLSX).

**Additional file 7:** **Table S7.** The genotype of 90 BC_1_ individuals from 2020 tested by KM2191098 (XLSX).

**Additional file 8:Table S8.** The primer sequences of KM2191098 (XLSX).

**Additional file 9:Figure S1.** Frequency distributions spinach leaf-related traits in BC_1_ population from 2019.

## Data Availability

The datasets generated and analyzed during the current study are available in the supplementary Tables.

## Abbreviations

AFLP: : Amplified fragment length polymorphism
GBS: Genotyping-by-sequencing
KASP: Kompetitive Allele Specific PCR
LL: Leaf length
LR: The ratio of leaf length to width
LW: Leaf width
MAS: Marker-assisted selection
PL: Petiole length
RAPD: Randomly amplified polymorphic DNA
RFLP: Restriction fragment length polymorphism
SLAF-seq: Specific locus amplified fragment sequencing
SNP: Single nucleotide polymorphism
SSR: Simple sequence repeats

## Declarations

**Ethical approval and consent to participate**.
