# Supplementary figures and images for: Quantitative trait loci (QTL) analysis of leaf related traits in spinach (Spinacia oleracea L.)
Source: BMC Plant Biol. 2021 Jun 24;21:290. doi: 10.1186/s12870-021-03092-5 (PMC8223354; doi:10.1186/s12870-021-03092-5)

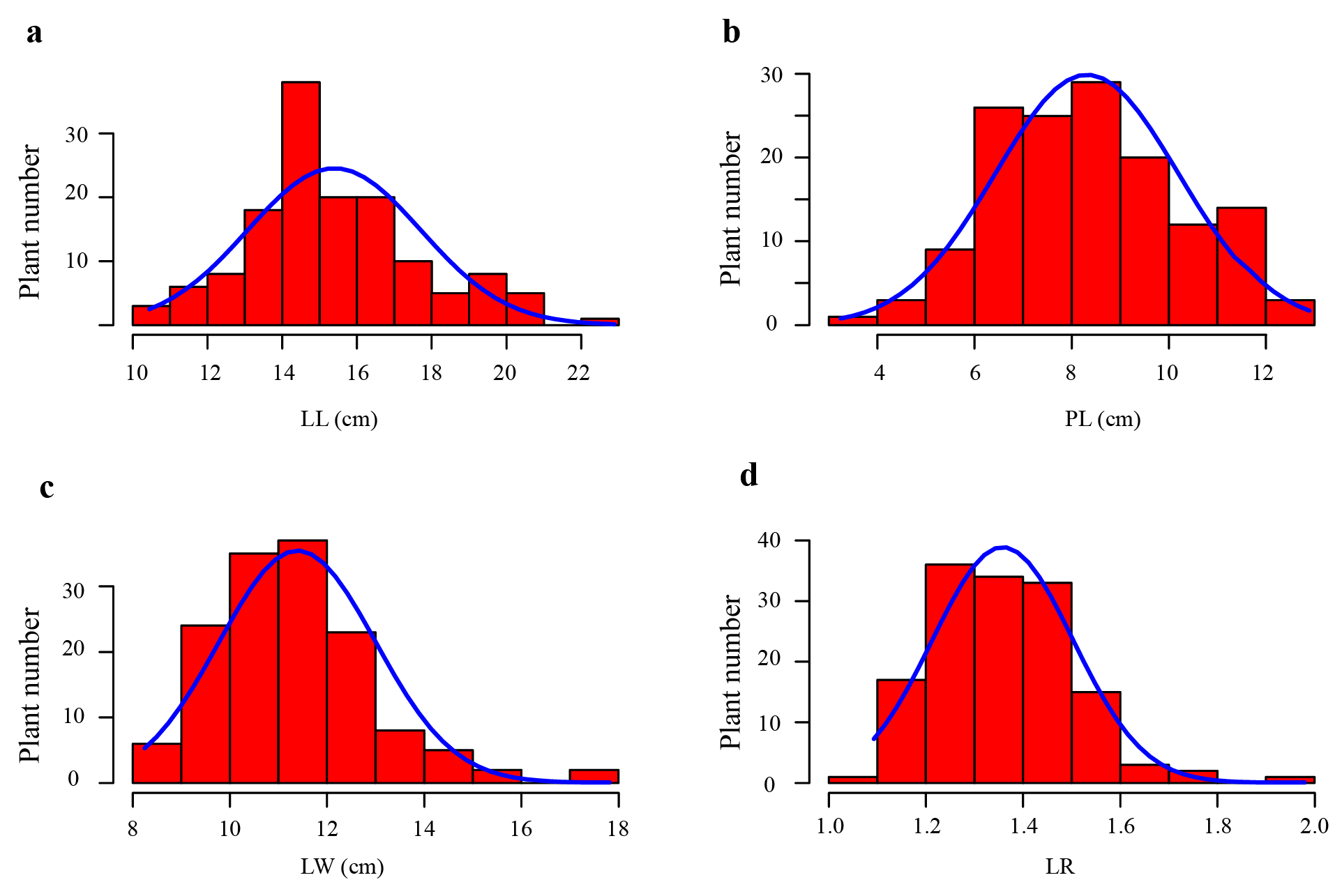

Supplement: Supplementary file 9 — Additional file 9:Figure S1. Frequency distributions spinach leaf-related traits in BC1 population from 2019. [file 12870_2021_3092_MOESM9_ESM.tif]
